# Supplementary material for: Comparative genomics provides new insights into the diversity, physiology, and sexuality of the only industrially exploited tremellomycete: Phaffia rhodozyma
Source: BMC Genomics. 2016 Nov 9;17:901. doi: 10.1186/s12864-016-3244-7 (PMC5103461; doi:10.1186/s12864-016-3244-7)
Supplement: Additional file 6: — List of orphan genes with links to PFAM (related to Additional file 1: Table S1). (ZIP 1428 kb) [file 12864_2016_3244_MOESM6_ESM.zip › PFAM_UNIQUE/G04824_P.html]

#                                                                            --- full sequence --- -------------- this domain -------------   hmm coord   ali coord   env coord
  
# target name        accession   tlen query name           accession   qlen   E-value  score  bias   #  of  c-Evalue  i-Evalue  score  bias  from    to  from    to  from    to  acc description of target
  
#------------------- ---------- ----- -------------------- ---------- ----- --------- ------ ----- --- --- --------- --------- ------ ----- ----- ----- ----- ----- ----- ----- ---- ---------------------
  
G04824\_P             -            339 Pro-rich\_19          PF15455.1    357      0.28    8.8   2.4   1   1   0.00041      0.82    7.3   2.4   225   276   153   210   120   218 0.65 - Proline-rich 19  
G04824\_P             -            339 TPX2                 PF06886.6     57     0.043   12.6   2.0   1   1     3e-05      0.18   10.6   2.0    23    57   275   309   260   309 0.90 - Targeting protein for Xklp2 (TPX2)
